# Early-stage resectable non-small cell lung cancer in Hungary

**DOI:** 10.3389/pore.2025.1612152

**Published:** 2025-07-28

**Authors:** Gabriella Gálffy, Réka Hécz, Réka Bujdosó, Eszter Gáspár, Réka Korompay, Judit Hoffer, Szilvia Szécsényi, Celia Blasszauer, Dániel Reibl, Erika Tóth, Krisztina Bogos, László Agócs, Ferenc Rényi-Vámos, Éva Mórocz

**Affiliations:** ^1^Department of Oncopulmonology, Pulmonology Center of the Reformed Church in Hungary, Törökbálint, Hungary; ^2^ AstraZeneca Ltd., Budapest, Hungary; ^3^ MedicalScan Ltd., Budapest, Hungary; ^4^Department of Surgical and Molecular Pathology, National Institute of Oncology, Budapest, Hungary; ^5^ National Korányi Institute of Pulmonology, Budapest, Hungary; ^6^Department of Thoracic Surgery, Semmelweis University, Budapest, Hungary; ^7^Department of Thoracic Surgery, National Institute of Oncology, Budapest, Hungary

**Keywords:** non-small cell lung cancer (NSCLC), epidemiology, early stage, curative surgery, EGFR mutation

## Abstract

This study provides a comprehensive analysis of early-stage resectable non-small cell lung cancer (NSCLC) in Hungary, investigating incidence rates, demographic trends, treatment patterns and survival outcomes. We used data from the National Health Insurance Fund (NHIF) spanning 2013–2022, and we analyzed 6,571 patients with available NSCLC histology and no metastasis, who underwent curative surgery within 6 months of diagnosis, and evaluated epidemiological trends and the use of neoadjuvant and adjuvant therapies. For the efficacy analysis, we narrowed the patient cohort to 5,494 patients diagnosed and treated between 2013 and 2019 with at least three-year follow-up data. Key endpoints included overall survival (OS) and disease-free survival (DFS), inferred via time to first subsequent therapy (TFST). Our results revealed a gradual decline in early-stage resectable NSCLC diagnoses, with a significant drop in 2020, likely linked to COVID-19 restrictions. Older age groups (66–75 years) represented a growing proportion of cases, reflecting shifting demographic trends. Among patients with EGFR mutations receiving EGFR tyrosine kinase inhibitor (EGFR-TKI) therapy, OS significantly improved compared to those not receiving EGFR-TKI therapy, who are assumed to have wild-type EGFR status (HR = 0.58 (95% CI: 0.47–0.72), p < 0.0001). These findings underscore the importance of early detection, comprehensive biomarker testing and targeted therapies in improving outcomes for resectable NSCLC patients. Future studies with extended follow-up and integration of broader clinical data, including staging and patient comorbidities, are warranted to optimize therapeutic strategies.

## Objective

The objective of this study was to determine overall survival (OS) and disease-free survival (DFS) in early-stage resectable non-small cell lung cancer (NSCLC) using the time to first subsequent therapy (TFST), an outcome of the comprehensive analysis of epidemiological data, patient care pathways, treatment patterns, and the efficacy of standard-of-care therapies for patients in Hungary from 2013 to 2022. This objective was outlined in a subpopulation of patients characterized by positive EGFR mutational status focusing on OS.

## Introduction

For many years, lung cancer was the most frequently diagnosed cancer and the leading cause of cancer-related death worldwide [1]. Although in 2020 female breast cancer has surpassed lung cancer as the most frequently diagnosed cancer (11.7% vs. 11.4%), lung cancer remained the leading cause of cancer death worldwide [2]. Non-small cell lung cancer (NSCLC) is the most common lung cancer, accounting for 80 to 85 percent of lung cancer cases, while small cell lung cancer (SCLC) makes up 15 to 20 percent [3, 4].

Non-metastatic NSCLC cases are categorized based on detailed locoregional staging. Early (ESMO guidelines I-IIIA) and locally advanced (ESMO guidelines IIIB/C) lung cancers are distinguished in the clinical practice. Patients diagnosed in early stages (I-IIIA) are candidates for surgical resection determined primarily by TNM staging and lymph node status, especially considering the N2 lymph node. Upon lymph node assessment, operability is determined based on cardiopulmonary status, comorbidities, and current pharmacological therapies. Lung cancers diagnosed at early stages (I-IIIA) have the potential of being cured by surgery alone or with neoadjuvant and/or adjuvant treatments [3, 5]. In the IB-IIIA resectable group, until recently, only chemotherapy was available as an adjuvant systemic treatment. However, EGFR-TKI and immuno-oncological adjuvant therapies are now also becoming available in Hungary. This patient group holds significant importance as they are eligible for innovative therapeutic options, which could provide substantial benefits through early screening and diagnosis. In Hungary this initiative is represented by the HUNCHEST nationwide screening program [6, 7].

There are no data sources in Hungary that contain all the necessary data required to identify early-stage resectable NSCLC cases in Hungary, therefore we have developed a unique methodology to identify this patient group.

In our real-world evidence study, we assessed the epidemiological data of the early-stage resectable NSCLC patient population and evaluated the disease-free survival and overall survival outcomes brought about by therapies accessible in the therapeutic setting of Hungary, prior to the advent of personalized therapies (2013–2022). The primary endpoints were DFS, inferred by TFST, and OS in patients who underwent curative surgery with a diagnosis consistent with NSCLC.

Previously, there was no data collected on early-stage lung cancer incidence in Hungary, there was only estimated data available [8]. The National Health Insurance Fund (NHIF) database on the entire lung cancer patient population–including SCLC and metastatic NSCLC - has been analyzed before [9–16], but we are the first to establish data for early-stage resectable NSCLC patients.

## Materials and methods

### Data source

This nationwide, retrospective, longitudinal study was conducted using data from the National Health Insurance Fund of Hungary (NHIF). The NHIF database is a national insurance system covering the Hungarian population almost entirely, which collects patient IDs, reimbursed prescriptions, a wide range of interventions and events, as well as ICD-10 (International Statistical Classification of Diseases, version ICD-10) coded records of all domestic in-patient and out-patient visits in Hungary. The NHIF also provides demographic data (age, gender, date of birth/death). In this study, we accessed the following sub-databases for further analysis: disease identification (ICD-10), diagnostics (DRG, Diagnosis Related Groups), and interventions (ICHI, International Classification of Health Interventions). The NHIF does not include certain data, such as EGFR mutation status, population-wide histology data, as it is not mandatory when reporting a therapy to the NHIF, and therapies used in clinical trials, since those are not funded by the social security system. There is no anamnestic history, e.g., smoking, available through NHIF either, as well as data on staging based on TNM classification, adverse events, ECOG status, lab results, and biomarker testing.

The National Health Research Ethics Council (BM/15360-1/2023) approved the study.

### Study population

**FIGURE 1:**
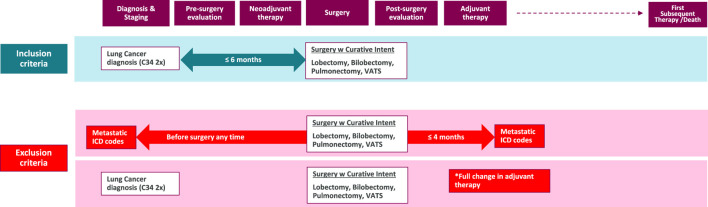
Inclusion and exclusion criteria for the study population. This flowchart illustrates the selection process of the study cohort. *Full change: The adjuvant therapy has been completely modified, leading us to suppose that, despite the curative intent of the surgery, disease progression has occurred in these patients, moving them beyond the early-stage category. ICD: International Classification of Diseases; w: with; VATS: Video-Assisted Thoracoscopic Surgery.

Patients were included if they were newly diagnosed with lung cancer (ICD-10: C34) between 1 January 2013, and 31 December 2022, aged 18 years or older at the time of diagnosis. To minimize the risk of misclassification, only patients with a second record of C34 ICD-10 code, documented no more than 12 months apart from the initial ICD code, were included. Patients with only a single C34 code who died within 2 months of the initial coding were also included to ensure accurate case capture. To filter for resectable cases, we narrowed down the population by surgical interventions with curative intent occurring within 6 months of diagnosis (Figure 1). We included patients in the analysis whose operability was confirmed 2013 onward. The procedures included are specific to NSCLC rather than SCLC and were determined as in the ADAURA clinical trial: lobectomy, bilobectomy, and pulmonectomy (and their subtypes) [17] (Figure 1).

Tumor histology data was furthermore utilized to confirm NSCLC cases by removing non-NSCLC diagnoses, when available. All data used in the analysis were anonymized, and only non-identifiable information was used throughout the investigation.

We defined neoadjuvant therapy as chemotherapy that was administered between diagnosis and surgery and adjuvant therapy as chemotherapy and/or radiotherapy within 4 months of surgery.

This outline was implemented as follows, reported in a cumulative manner (patient populations were determined yearly for analysis):

1. Between 2013 and 2022, 92,162 patients were found with any lung cancer (LC) diagnosis.
2. Of those patients, 20,824 had a history of thoracic surgery within the mentioned timeframe.
3. Based on the ADAURA criteria, 13,392 patients underwent curative surgery.
4. 9,896 patients met the eligibility criteria of having surgery no later than 6 months after their lung cancer diagnosis and had no documentation of metastasis any time before and 4 months after surgery. This patient group represents the overall incidence of early-stage, resectable NSCLC cases observed during the study period.
5. NSCLC-associated histology was confirmed in 6,571 patients (histology was not available in numerous cases as it is not a mandatory requirement for insurance claims related to diagnostic or therapeutic procedures. The histological type of the tumor was recorded in 66% (6,571/9,896) of the early-stage resectable NSCLC cases. We analyzed epidemiology data, adjuvant and neoadjuvant therapy use in this population.
6. According to our analysis, three-year follow-up data was required, meaning patients recorded between 2013–2019, as such, 5,494 patients made up our final study population for efficacy analysis.

### Statistical analysis

To analyze clinical outcomes, specifically overall survival (OS) and DFS (estimated by time to first subsequent therapy (TFST) or death). We used ‘survival’ and ‘survminer’ packages in R (version 4.2.1) for Cox regression and Kaplan-Meier analysis. Kaplan-Meier analysis as a non-parametric statistic is used to estimate the survival function from lifetime data, particularly useful in medical research for analyzing the time until an event of interest, such as death or recurrence of a disease. The Kaplan-Meier estimator provided the probability of surviving a given length of time while considering the occurrence of specific events (e.g., death or relapse).

Cox regression, or Cox proportional hazards model, is a statistical method used to analyze the effect of variables on the time until an event occurs, assuming proportional hazard ratios over time.

## Results

Epidemiological analysis was performed on the 6,571 patients with confirmed histology of NSCLC, while 5,494 patients were selected (data availability) for our follow-up analysis 3 years after undergoing first curative-intent treatment for early-stage NSCLC. 3,151 either died or received first subsequent therapy, indicating disease progression, while 2,343 lived during the full study period without the administration of first subsequent therapy. 2,298, out of 3,151 progressing patients received subsequent therapy or died within 3 years. By the end of the 3^rd^ year, 3,837 patients were still alive, and 3,200 patients lived without disease progression.

### Cohort characteristics

**FIGURE 2:**
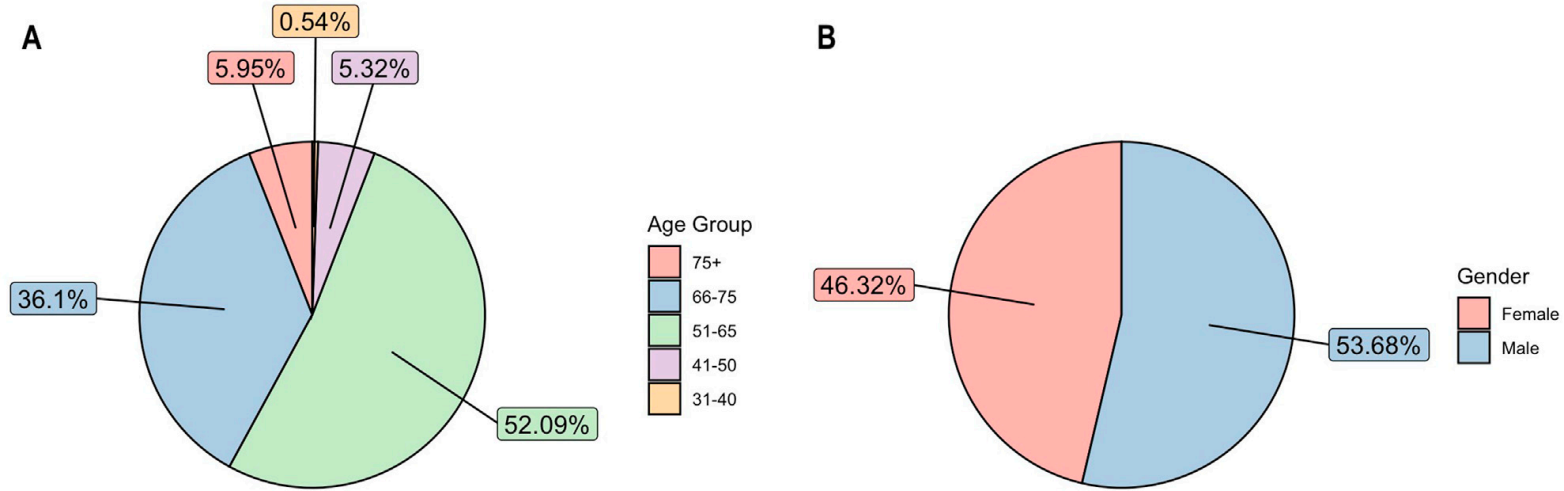
**(A)** Average age distribution among patients over study period 2013–2022. **(B)** Average gender distribution among patients over study period 2013–2022.

In terms of age, the incidence of early-stage operable NSCLC patients with confirmed histology diagnosed between 2013 and 2022 (N = 6,571), most patients having been diagnosed at 51–65 years of age (Figure 2). Over the study period, however, the proportion gradually shifted towards the older age group. The proportion of patients aged between 51 and 65 decreased (60.26% in 2013 and only 40.71% in 2022), while the proportion of people aged between 66 and 75 increased (29.77% in 2013 and 47.81% in 2022). According to lung cancer reports, the greatest decrease was found among the younger age group (40–49 to 50–59 years), independent of sex. In contrast, the incidence decreased less in the 60–69 and 70–79 age groups.

Between 2013 and 2022, operable NSCLC was more common in men than in women (53.68% vs. 46.32%) (Figure 2). Although lung cancer continues to be more prevalent in men than in women, this gap is gradually narrowing (male-to-female ratio: 54.51% vs. 45.49% in 2013; 51.19% vs. 48.81% in 2022) in line with both international and domestic trends. Data from the Korányi Bulletin [18] (Korányi Institute in Hungary) show an even more pronounced change among all lung cancer patients in the pulmonary care system (male-to-female ratio in 1980, 84% vs. 16%; in 2013, 61% vs. 39%; in 2022, 53% vs. 47%).

### Real-world evidence on treatment approaches

According to our study design, treatment involves curative surgery ± neoadjuvant (chemotherapy) and/or adjuvant (radiotherapy, chemotherapy) therapy. These interventions do not include the therapies used in clinical trials, since those are not funded by the social security system and therefore not included in the NHIF database.

Among patients with at least two C34 codes between 2013 and 2022 (N = 92,162), 20,824 patients (22.59%) underwent any type of thoracic surgery. Of these, 13,392 patients (64%) underwent surgeries classified as curative, such as Video-Assisted Thoracoscopic Surgery (VATS) anatomical resection, lobectomy, bilobectomy, pneumonectomy, or sleeve resection. Curative surgeries were performed on 14.5% (13,392/92,162) of all lung cancer patients with two C34 codes in the period from 2013 to 2022. In 2022 these percentages were higher than the period average; 24.5% (1,768/7,218) of patients with two C34 codes underwent any type of thoracic surgery for lung cancer, and curative surgery was performed in 15.3% (1,102/7,218) of cases. A potential explanation for the higher percentages of all thoracic surgeries and curative surgeries in 2022 is a shift from the COVID years, when surgeries were often postponed. This assumption is supported by the increase in the number of neoadjuvant therapies during 2020–2021.

Between 2013 and 2022, the average time from diagnosis (marked by the first C34 code) to the day of surgery was 53 days, with a median of 47 days, in the analyzed patient group (N = 6,571). However, during 2021–2022, the average time increased to 61 days (median 55) in 2021 and 63 days (median 58) in 2022. This increase is presumably attributed to delays related to the COVID-19 pandemic. When neoadjuvant therapy preceded surgery, the time to first treatment was shorter, averaging 36.6 days, with a median of 35 days for the entire period.

**FIGURE 3:**
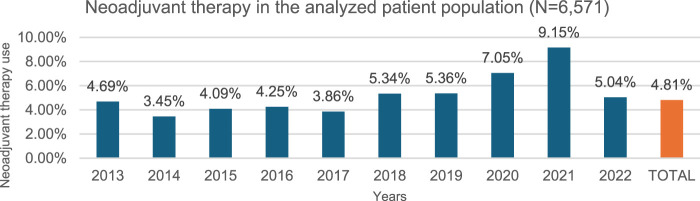
Neoadjuvant therapy use per year. 4.81% of the study population (N = 6,571) (over 18y, 2x C34 within 12 months, curative surgery within 6 months of diagnosis, NSCLC histology, no metastasis) received neoadjuvant chemotherapy preceding surgery as first treatment during the study period of 2013–2022.

**FIGURE 4:**
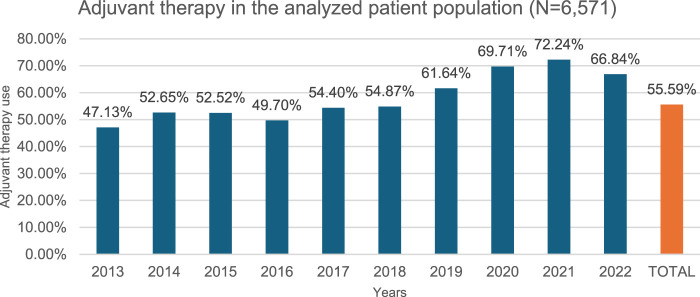
Adjuvant therapy use per year. 55.59% of the study population (N = 6,571) (over 18y, 2x C34 within 12 months, curative surgery within 6 months of diagnosis, NSCLC histology, no metastasis) received adjuvant chemotherapy or radiotherapy following surgery, meaning disease progression during the study period of 2013–2022.

In our study, 316 of 6,571 patients received neoadjuvant therapy between 2013 and 2022 at a constant rate over the years (Figure 3), meanwhile 3,653 patients received adjuvant therapy out of 6,571 total patients in the epidemiological analysis group between 2013 and 2022, at a steadily increasing rate (Figure 4).

Neoadjuvant therapy: Since neoadjuvant chemotherapy offers similar benefits to adjuvant chemotherapy but may complicate surgery, ESMO guideline recommend adjuvant chemotherapy over neoadjuvant chemotherapy [19]. Our data confirm a similar trend in Hungary, and in our patient group analyzed for epidemiology data (N = 6,571), 4.81% (316/6,571) received pre-operative neoadjuvant treatment between 2013 and 2022, with little variation over time (4.69% in 2013, 5.04% in 2022), except for an increase in 2020 (7.05%) and 2021 (9.15%) due to COVID-related surgical delays (Figure 3).

Adjuvant therapy: In our study, 55.59% (3,653/6,571) of patients who underwent curative surgery were adjuvant therapy (chemotherapy, radiotherapy) recipients. This rate showed a steady increase over the period of 2013–2022, with the proportion rising from 47.3% in 2013 to 66.84% in 2022 (402/853 vs. 252/377) (Figure 4). This is consistent with data from the ADAURA study [10]. In our study, 71% (2,597/3,653) of patients received adjuvant therapy in the second month, 18% in the third month (673/3,653).

## Efficacy results

The efficacy of the curative surgery ± neoadjuvant and/or adjuvant therapy was evaluated in a strongly restricted patient group given a minimum follow-up period of 3 years; we analyzed data from 5,494 patients diagnosed between 2013 and 2019 in Hungary.

### Disease-free survival (DFS)

**FIGURE 5:**
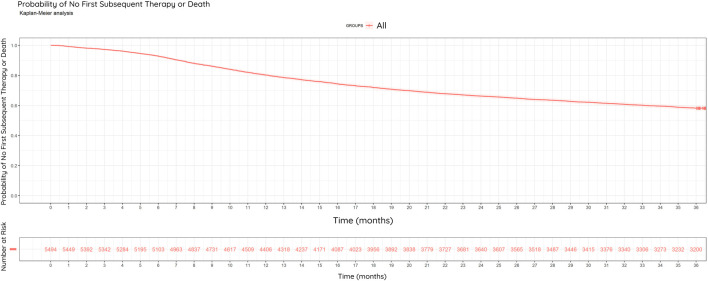
Kaplan-Meier Curve: Disease-Free Survival (DFS). Probability of no first subsequent therapy or death among the restricted study population (N = 5,494) between 2013–2022, within 3 years of surgery. Estimated DFS rate at the end of year three was 58.2%.

Of the 5,494 patients, 3,151 (57.3%) received first subsequent therapy or died during the full study period, while 2,343 patients (42.7%) did not receive subsequent therapy and were alive. In the study population, 41.8% (2,294/5,494) of all patients and 72.8% (2,294/3,151) of progressing patients received subsequent therapy or died within 3 years. Based on the number of patients not administered subsequent therapy, the estimated DFS rate at the end of the 3^rd^ year is 58.2% (3,200/5,494) (Figure 5).

In our study, 29% of patients had developed metastases (1,583/5,494). 77% of the metastases found in our study (22% of all surgical patients) were formed within 3 years of surgery.

### Overall survival (OS)

**FIGURE 6:**
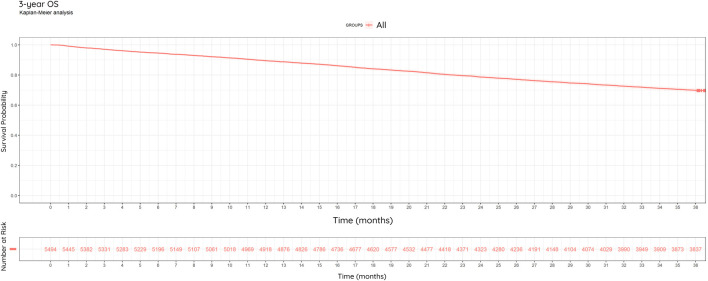
Kaplan-Meier Curve: Overall Survival (OS). 3-year overall survival of study population (N = 5,494) between 2013–2022. 69.8% of patients were alive at the end of the observation period.

Of the 5,494 patients, 69.8% (3,837/5,494) were alive at the end of year 3% and 30.2% (1,657/5,495) were dead (Figure 6).

**FIGURE 7:**
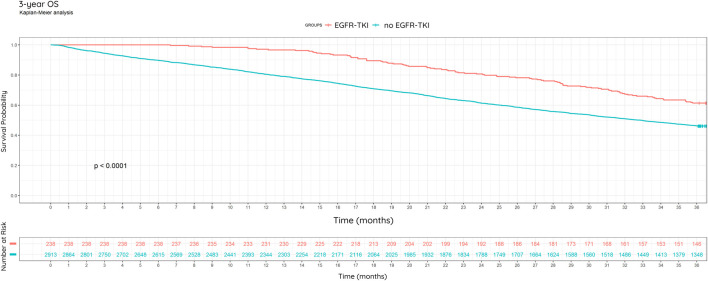
3-year overall survival, including EGFR mutational status and treatment. Kaplan-Meier analysis.

We analyzed the 3-year OS in subgroups of progressing patients who received EGFR-TKI therapy due to EGFR mutation positivity and those who likely did not receive EGFR-TKI therapy due to negative EGFR mutation status (Figure 7).

Among progressing patients, there is some information on the nature of follow-up therapy. In our study, subgroups of patients receiving EGFR-TKI (gefitinib, erlotinib, afatinib) in any line due to EGFR mutation (EGFRm) positivity, and not receiving EGFR-TKI due to assumable mutation negativity, were identified. EGFR testing results were not available to us through NHIF, instead we confirmed EGFRm positive status through documented EGFR-TKI treatment, while EGFR wild-type status could not be ascertained as there may have been cases where EGFRm positive patients were left untreated. Of the 3,151 progressing patients, 238 (7.55%) received first- or second-generation EGFR-TKIs, while 92.5% received other treatments (chemotherapy or immunotherapy). This rate is slightly lower than the rate of EGFR mutation (10%–15%) in NSCLC in the Caucasian population [20]. During the investigated period, EGFR testing was not routinely performed in all patients. The implementation of EGFR testing gradually increased over time, becoming standard practice.

Based on the Kaplan-Meier curve of patients with EGFRm positive NSCLC, there was an enhanced survival in this population compared to patients suspected to be non-EGFRm positive (Kaplan-Meier, percentage: p < 0.0001) (Figure 7).

In the subgroup of EGFR mutation-positive NSCLC patients, 61.3% (146/238) were alive at the end of year 3. In the subgroup of 2,913 presumably EGFR mutation-negative patients with progressing non-small cell lung cancer who did not receive EGFR-TKI therapy, 46.3% (1,348/2,913) were alive at the end of year 3. In the EGFR-TKI group, median survival is not reached, while in the non-EGFR-TKI group it is 1000 days (95% CI: 953–1044 days). Based on Cox regression, EGFR-TKI treatment is associated with significantly better survival (HR = 0.58, 95% CI: 0.47–0.72, p < 0.0001) (Figure 7).

## Discussion

To preface this section, it is important to highlight that incidence was analyzed in a cohort of 9,896 patients between 2013 and 2022. For subsequent analyses, we included only patients with available histology results, totaling 6,761 individuals. Demographic data and the use of adjuvant/neoadjuvant therapy were examined in this subset of patients. The strongly restricted patient population of 5,494 with three-year follow-up data available (2013–2019) underwent efficacy analyses.

If we had the accurate annual number of lung cancer diagnoses in Hungary, along with reliable histological results, we could determine the incidence of NSCLC patients. By integrating staging data for early stages (I-IIIa) based on the TNM classification used in clinical practice, we could identify the early-stage resectable NSCLC patient group. Unfortunately, no database in Hungary contains all these data. Therefore, we have developed a unique methodology to identify the early-stage NSCLC patient group by analyzing NHIF claims database.

Every year, the largest pulmonology center of Hungary, the Korányi Institute, publishes data on patients admitted to pulmonary healthcare facilities. As such it has a database covering 60% of lung cancer cases nationwide. The limitation of their publication is that nationwide data can only be obtained by extrapolation. Other attempts to define patient cohorts have also been made, albeit not regarding resectable NSCLC [9–16].

The quality of a claims database is heavily dependent on reporting disciplines and variables of coding options and routines, accounting for the differences between the databases. The NHIF database includes all events and interventions, however there may be regional differences in coding protocols.

Our analysis is reinforced by the fact that we analyzed a national database covering practically every Hungarian citizen, which allowed a large number of patients to be identified. On the other hand, detailed anamnestic history is not available in this database (e.g., smoking). There are no data on TNM staging (meaning operable IIIB cases were involved in our study), adverse events, ECOG status and lab results, or biomarker testing. Furthermore, histological diagnosis is not available for all patients.

It would greatly improve the accuracy of the data if the updated SNOMED CT (Systematized Nomenclature of Medicine Clinical Terms) codes were included in the NHIF database in addition to or instead of the ICD codes. SNOMED CT is internationally recognized and used in various countries around the world. Its global adoption helps standardize healthcare terminology across borders, which is especially beneficial for national and multinational healthcare organizations and for managing health data in global public health initiatives [21].

The patient population in our analysis included patients who passed multiple filters between 2013 and 2019: aged 18 years and older, either gender, had two ICD-10 C34 coded (lung cancer) diagnoses within 12 months of each other (92,162 patients), underwent curative surgery within 6 months of the first lung cancer diagnosis with no documented metastasis any time before or 4 months after the operation (9,896 patients), had histology results confirming NSCLC (6,571 patients), and had three-year follow-up data, meaning latest data is from 2022 (5,494 patients).

Based on the NHIF morphology report, lung cancer histology typing was recorded in 66% of the early-stage resectable NSCLC patients. A separate Hungarian real-world evidence study, examining the entire lung cancer population, reported availability of histology reports in 56.3% of all lung cancer patients [15].

According to the NHIF database, the number of C34 coded (twice within a 12-month period) lung cancer patients gradually decreased between 2013 and 2022: 10,514 patients in 2013 compared to 7,218 patients in 2022. This decline is similarly reflected in the nationwide data of the Korányi Bulletin of 2023, where 5,415 patients in 2013 contrasted 3,397 patients in 2022 [11, 18].

The national cancer registry could potentially overestimate the number of lung cancer cases, and recent publications have highlighted its limitations concerning the reliability of tumor classification [11, 22, 23].

In a Danish real-world evidence study on early-stage NSCLC from 2013–2018 (N = 8,758), patients were most frequently diagnosed above 70 years of age (55.5% of patients), with only 13% of patients being under 60 years of age [24], whereas in our study, most patients were diagnosed between 51 and 75 years of age. Although, our proportion gradually shifted towards the older age group over the period of study. In terms of gender, the Danish real-world evidence study also found similarly to us that the diagnosed early-stage NSCLC patients between 2013 and 2018, were nearly equal proportions of men and women (49.2% vs. 50.8%) [24]. In an Italian real-life data analysis in a reference thoracic oncology unit (N = 225), most early-stage NSCLC patients were men (57% vs. 43%) [25], while in a US real-life data analysis (N = 703) the majority of early-stage NSCLC patients were women (55.8% vs. 44.2%) [26]. However, it is worth noting that as almost all cases of early-stage NSCLC are usually defined as resectable, the terms “early-stage resectable” and “early-stage” are used interchangeably in certain publications.

Regarding treatment protocols, according to the Hungarian Korányi Bulletin 2023 [18], 8% of patients operated on in 2022 received neoadjuvant chemotherapy, 31.3% received adjuvant chemotherapy, and 7.4% received both chemo- and radiotherapy. Data from the ADAURA study, where investigators were able to decide whether to administer adjuvant chemotherapy to patients based on clinical experience in NSCLC of stage IB-IIIA, were comparable. In the ADAURA trial, 60% of patients who underwent curative surgery and were resected to R0 received adjuvant chemotherapy [17]. In an Italian real-life study, 18% of patients received adjuvant chemotherapy (41/225) [25]. In our study, 55.59% (3,653/6,571) of patients who underwent curative surgery were adjuvant therapy (chemotherapy, radiotherapy) recipients.

Regarding metastases, we found that 29% of patients had developed them (1,583/5,494). This can be compared to the ADAURA study, where 28% of patients in the control arm had metastases [17].

In cases where surgery was the first treatment, it took an average of 53.63 days (with a median of 47 days) from diagnosis (defined as the first appearance of C34) to undergo surgery. In contrast, when neoadjuvant therapy preceded surgery, the time to first treatment was shorter, averaging 36.6 days (median of 35 days). A separate Hungarian real-world evidence study, examining the entire lung cancer population, reported a median treatment interval (time between first C34 code and start of therapy) of 37.83 days for adenocarcinoma and similarly for squamous cell carcinoma [16].

Shifting focus to the topic of EGFR mutation, gefitinib was the first registered treatment with EGFR-TKI for lung cancer in 2009, specifically for EGFR mutation positive, locally advanced, and metastatic non-small cell lung cancer. This therapy has been available in Hungary since 2011 for metastatic adenocarcinoma. While the efficacy and safety of the therapy was demonstrated in a first line setting in a pivotal clinical trial, early real-world use in Hungary was pushed back to later lines due to limitations in EGFR mutation testing. Nowadays, EGFR mutation testing is routine in all metastatic NSCLCs and attempts are underway to make it routine at an early stage. Our results underscore the significant impact of targeted therapies, such as EGFR-TKIs, on improving overall survival in patients with EGFR mutation-positive NSCLC, highlighting the importance of molecular diagnostics in guiding treatment decisions.

In the progressing patient population, 7.55% received first- or second-generation EGFR-TKIs in any line during the study period (2013–2022). EGFR testing was not standard practice for all patients during the study period, with its implementation gradually increasing over time. As mentioned before, a mere 3% of patients were already receiving first-line targeted EGFR-TKI therapy when the observation period started, and it has grown more widespread since. This 3% rate also acts as a testing positivity rate, as not every patient had tests performed. Given that EGFR mutation testing has therapeutic implications even at an early stage, routine testing has become essential in adenocarcinoma at an early stage.

To summarize our findings, this study provides a comprehensive analysis of the epidemiology, treatment patterns and the efficacy of standard-of-care therapies for NSCLC in Hungary over the past decade. The data reveal a gradual decline in the incidence of resectable early-stage lung cancer and a shift in age distribution towards older patients. The use of adjuvant therapies, particularly chemotherapy, has steadily increased over time, in line with international treatment trends. However, neoadjuvant therapy remains underutilized, in line with current recommendations favoring adjuvant over neoadjuvant approaches.

The study confirmed that the prognosis of patients with known EGFR-TKI mutations is better than that of the wild type. With longer-term follow-up, further conclusions can be drawn in this patient group.

The higher overall survival observed in the EGFR mutation-positive subgroup is likely consequent to the availability of targeted therapeutic options (EGFR-TKI treatment) during this period. In contrast, until 2017, no personalized immuno-oncological or targeted therapies were available for the treatment of EGFR wild-type tumors, except for ALK mutation-positive tumors, which occur in approximately 4% of NSCLC cases. Since the advent of the first EGFR-TKIs as of 2022, molecular diagnosis has been routine practice in major centers, and the Hungarian Molecular Pathology Professional Directive, first published in 2024, includes EGFR reflex testing as a recommendation [27].

## Conclusion

There are prospects of alleviating disease burden that requires interdisciplinary collaborations. Due to the complexity of this disease regarding staging and therapeutic decision-making, patients and practitioners alike benefit from establishing multidisciplinary teams consisting of pulmonary oncologists, thoracic surgeons, bronchologists, molecular pathologists, and radiation oncologists. Our analysis not only provides real-world evidence of current resectable NSCLC patient outcomes but also suggests important points to clinicians and policymakers.

1. The development of national registries, incorporating updated SNOMED codes into the NHIF database—in addition to or replacing BNO codes—and harmonizing coding practices in Hungary would enhance the understanding of real-world patient data and enable more detailed analyses of patient outcomes.
2. Early detection of lung cancer has a huge impact on patient outcomes, therefore early screening of lung cancers should also be implemented, such as the HUNCHEST screening program [6, 7].
3. Also due to reflex-testing in early-stage NSCLC in Hungary, increased number of patients have been identified who can benefit from modern targeted therapies.

Our foundational analysis provides a base for future comparisons in the real-world setting to investigate how new therapies may impact patient outcomes.

## Data Availability

The original contributions presented in the study are included in the article/supplementary material, further inquiries can be directed to the corresponding author.
